# Secreted Cysteine-Rich Repeat Proteins “SCREPs”: A Novel Multi-Domain Architecture

**DOI:** 10.3389/fphar.2018.01333

**Published:** 2018-11-20

**Authors:** Michael Maxwell, Eivind A. B. Undheim, Mehdi Mobli

**Affiliations:** Centre for Advanced Imaging, The University of Queensland, St Lucia, QLD, Australia

**Keywords:** peptide, tandem repeat, toxin, ion channel, disulfide rich, venom, SCREP

## Abstract

Peptide toxins isolated from animal venom secretions have proven to be useful pharmacological tools for probing the structure and function of a number of molecular receptors. Their molecular structures are stabilized by posttranslational formation of multiple disulfide bonds formed between sidechain thiols of cysteine residues, resulting in high thermal and chemical stability. Many of these peptides have been found to be potent modulators of ion channels, making them particularly influential in this field. Recently, several peptide toxins have been described that have an unusual tandem repeat organization, while also eliciting a unique pharmacological response toward ion channels. Most of these are two-domain peptide toxins from spider venoms, such as the double-knot toxin (DkTx), isolated from the Earth Tiger tarantula (*Haplopelma schmidti*). The unusual pharmacology of DkTx is its high avidity for its receptor (TRPV1), a property that has been attributed to a bivalent mode-of-action. DkTx has subsequently proven a powerful tool for elucidating the structural basis for the function of the TRPV1 channel. Interestingly, all tandem repeat peptides functionally characterized to date share this high avidity to their respective binding targets, suggesting they comprise an unrecognized structural class of peptides with unique structural features that result in a characteristic set of pharmacological properties. In this article, we explore the prevalence of this emerging class of peptides, which we have named Secreted, Cysteine-rich REpeat Peptides, or “SCREPs.” To achieve this, we have employed data mining techniques to extract SCREP-like sequences from the UniProtKB database, yielding approximately sixty thousand candidates. These results indicate that SCREPs exist within a diverse range of species with greatly varying sizes and predicted fold types, and likely include peptides with novel structures and unique modes of action. We present our approach to mining this database for discovery of novel ion-channel modulators and discuss a number of “hits” as promising leads for further investigation. Our database of SCREPs thus constitutes a novel resource for biodiscovery and highlights the value of a data-driven approach to the identification of new bioactive pharmacological tools and therapeutic lead molecules.

## Introduction

The need for more advanced therapeutics has driven the recent growth in interest toward the use of natural sources in drug discovery and development programs (Newman and Cragg, 2016). A major natural source of secreted proteins that can selectively modulate ion-channel activity with high potency, are small toxins (<100 residues) found within the secretions of animal venoms as a heterogeneous mixture of compounds (Lavergne et al., 2015). A common distinctive feature of these toxins is their cysteine-rich nature, resulting in a disulfide framework with a diverse range of specific connectivity patterns. Here, we outline many variations of such disulfide-stabilized protein folds, collectively referred to as disulfide-rich domains (DRDs). One approach toward structural classification of DRDs, is by clustering the spatial arrangement of secondary structural features. Previously this has led to the identification of 41 unique fold groups, identified from 963 domain representatives (Cheek et al., 2006). Within these major fold groups exist numerous examples of venom derived DRDs. Some major domain types found within toxins throughout a diverse taxonomic range include: the inhibitor cystine knot (ICK) which is included among the “knottins” (Gracy et al., 2008), the Kunitz/Bovine pancreatic trypsin inhibitor (BPTI) (Ascenzi et al., 2003), the Kazal-like domain (Friedrich et al., 1993), the whey acidic protein (WAP) domain (Hennighausen and Sippel, 1982), the ShKT-like domain (Castaneda et al., 1995), and the phospholipase A2 (PLA_2_) enzymatic domain (Rigoni et al., 2005; Lavergne et al., 2015). The vast majority of previously studied toxins contain a *single* independently folded region of a specific domain type, such as taicatoxin (TCX) which consists of a 50 residue long BPTI/Kunitz inhibitor domain supported with three disulfide bonds (Possani et al., 1992), or Huwentoxin-IV which is a spider toxin with an ICK motif of 34 residues in length with three stabilizing disulfides (Peng et al., 2002).

Recently, a number of two-domain toxins with a tandem repeat (TR) architecture have been observed, highlighting a unique deviation from the standard single domain toxin architecture. The existence of evolutionarily formed, two-domain toxins with bivalent activity, was first observed in Rhodniin; a highly specific serine protease inhibitor with two Kazal-type domains, isolated from *Rhodnius prolixus* (van de Locht et al., 1995). More recently, the investigation of both τ-theraphotoxin-Hs1a (DkTx) (Bohlen et al., 2010) and π-hexatoxin-Hi1a (Hi1a) (Chassagnon et al., 2017), reveals that this novel TR architecture provides these toxins with an unusually high avidity for their ion-channel targets, which is proposed to be due to a bivalent interaction mechanism. Here, we focus on the presence of additional examples of such TR toxins, as well as defining a broader population of naturally occurring secreted, cysteine-rich repeat proteins (SCREPs), to which the TR toxins belong. An emphasis is placed on a data driven approach toward the identification and analysis of this population, and we describe the necessary tools and resources that we used in guiding the identification of bioactive SCREPs. Our results reveal that SCREPs comprise a surprisingly high number of diverse protein sequences, a portion of which display high levels of sequence identity with previously studied ion-channel impairing toxins.

## A Data Driven Approach in Protein Bio-Discovery

The protein “universe,” that is, the total space of all proteins from every species, consists of a vast and complex network of biomolecules. The rise of next-generation sequencing technologies has provided an excellent platform in which to explore this space. At first glance, the observable protein landscape appears disordered and random. However, upon further analysis, patterns of increasingly well-defined clusters are beginning to emerge. A growing number of detailed protein classifications now exist, with many groups branching from smaller central points; defined broadly by their structural similarities (Ladunga, 1992). Due to the overwhelming size and rapid accumulation of sequence data, the use of bioinformatics is essential in delineating protein relationships. Currently, most protein sequences exist without meaningful annotations and represent a wealth of unexplored territory. It is within this region that we investigate a novel architecture of secreted proteins, focusing on poorly defined toxin-like sequences that possess the potential for unique ion-channel pharmacology (Mobli et al., 2017).

A central process observed between all domains of life, is the presence of proteins containing a signal sequence region; usually 1–30 amino acid residues (aa’s hereafter) N-terminally located, that determine its cellular and subcellular locations (Pohlschroder et al., 1997). There exists a significant amount of variation between the transmembrane proteins that selectively process signal regions, translocating mature proteins across the target membrane (Muller, 1992). The term “secreted” is often ambiguously defined, however, within the context of this work, “secreted” refers to proteins destined for *extracellular* translocation. The purpose for this distinction is to help guide the identification of highly stable protein ligands that interact with extracellularly located receptors. Our ability to isolate and study these interactions may provide vital clues in the development of more sophisticated therapeutics needed for the treatment of complex pathophysiological disorders. Few disorders parallel in complexity to those associated with impaired ion-channel function; termed ‘channelopathies.’ Due to the fundamental physiological role of ion-channel activity, impairment can result in dramatic dysregulation of primary functions, affecting tissues such as cardiac muscle (Marban, 2002), skeletal muscle (Cannon, 2006), as well as physiological processes such as nociception (Bennett and Woods, 2014) and regulation of immune responses (Chandy and Norton, 2017). Therefore, the discovery of new tools enabling the selective study of subtype specific ion-channel activity has the potential to greatly assist in the rational design of new therapeutics.

## Protein Targets of Known Screps

There have been few studies of the pharmacology of SCREPs, however, those that have received attention reveal unique bivalent interactions and involve two major types of molecular targets, namely serine proteases and ion channels. As mentioned above, the first investigated SCREP was the selective thrombin-type serine protease inhibiting rhodniin (van de Locht et al., 1995). A similar SCREP later emerged, this time isolated from *Boophilus microplus*. Boophilin revealed a double Kunitz-type protease inhibitor architecture with a possible bi-functional role, inhibiting both circulating thrombin as well as the membrane bound thrombin activation intermediate, meizothrombin (Macedo-Ribeiro et al., 2008). This later study suggests there may be a potential role of bivalent disulfide rich peptide (DRP) interactions with membrane bound proteins. Even greater evidence supporting this notion was discovered after investigation of CpTx-1; a double-ICK peptide from the venom of the spider *Cheiracanthium punctorium.* This two-domain toxin had an observed effect on the resting membrane potential *and* a positive shift in α-helical structure composition in bicelle suspension (Vassilevski et al., 2010). These intriguing results indicate a possible interaction between CpTx-1 and both ion channels and the surrounding lipid membrane environment, a mechanism that has been shown to be important in many other toxins (Zhang et al., 2018). The specific interaction of SCREPs with ion channels has recently been demonstrated in two additional examples; DkTx and Hi1a, both of which represent a new role of bivalency driven ion-channel modulation.

**FIGURE 1:**
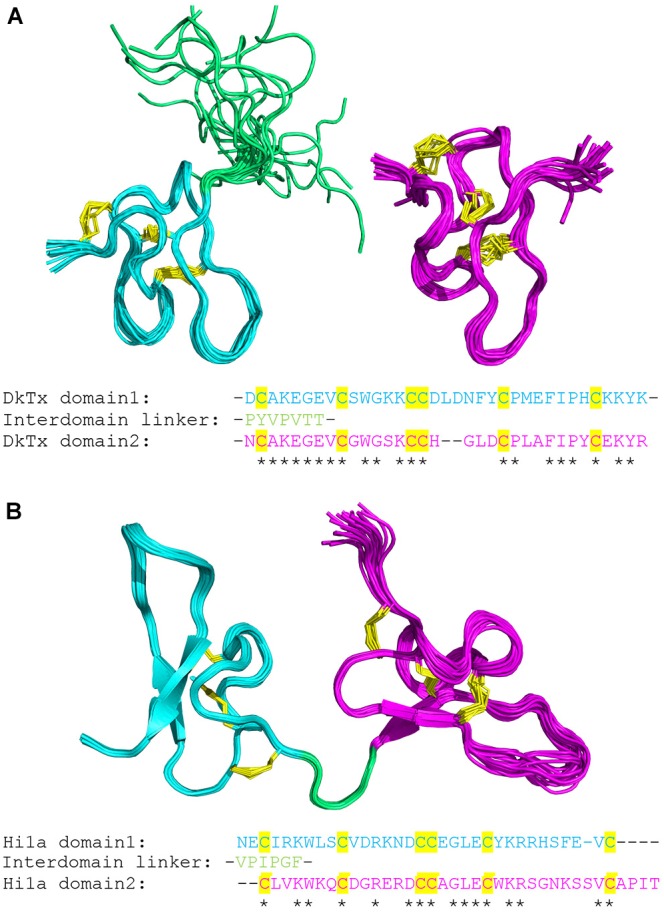
3D structure and domain alignment of ion-channel impairing SCREPs. **(A)** Solution structure of each individual domain of DkTx, with D1 (PDB-2N9Z) and D2 (PDB-2NAJ) in cyan and purple, respectively. The disulfide framework is highlighted in yellow, and the highly flexible linker region is shown in green. Below the structure is the primary sequence alignment of both domains, demonstrating the high level of homology between domains. **(B)** Solution structure of Hi1a (PDB-2N8F), highlighting each individual disulfide-rich domain connected by a more rigid peptide linker region. The aligned sequences of the individual domains are shown below.

DkTx was isolated from the Earth Tiger tarantula (*Haplopelma schmidti*), contains two independently folded ICK domains connected by a linker (Bohlen et al., 2010), and is a highly selective agonist of the TRPV1 channel, displaying no apparent effect when applied to HEK293 cells expressing a range of other ion channels (Bohlen et al., 2010). As seen in Figure 1A, each domain within the primary sequence self-aligns, demonstrating its TR nature. Both domains of DkTx appear to interact with the outer pore region of TRPV1, favoring the open channel state; loops 2 and 4 engage with the S6 helix on adjacent subunits, suggesting that DkTx acts as an allosteric pore modulator (Bae et al., 2016). The bivalent nature of this interaction forms a very stable complex, resulting in extremely high avidity. Recent structural studies of the TRPV1-DkTx complex using cryo-electron microscopy and NMR derived restraints, revealed that two DkTx peptides may interact with one TRPV1 tetramer (Gao et al., 2016). Overall, DkTx acts as a virtually irreversible agonist, binding to TRPV1 in the open state and preventing conformational return to the resting, closed, state.

More recently, another SCREP, π-hexatoxin-Hi1a (Hi1a), a 75-residue peptide isolated from the Australian funnel-web spider (*Hadronyche infensa*), demonstrated great potential for neuroprotection from stroke induced cerebral ischemia within a rat model (Chassagnon et al., 2017). Hi1a partially inhibits the activation of acid-sensing ion channel 1a (ASIC1a), an important ion channel involved in acidosis-induced neuronal damage in the mammalian brain (Li et al., 2010; Wemmie et al., 2003). Hi1a is again composed of two homologous ICK domains (Figure 1B), however, in contrast to DkTx the domains are connected by a much shorter and structurally well-defined linker. Like DkTx, unique activity is observed when testing the domains individually. The N-terminal domain retains activity in isolation, but with reduced potency and full channel inhibition. The C-terminal domain displays no apparent inhibition, however, when comparing the activity of the single N-terminal domain with the full length Hi1a, the difference in potency and altered level of inhibition indicates the existence of an underlying bivalent interaction (Chassagnon et al., 2017). Overall, the two-domain structure of these SCREPs demonstrate a very interesting antibody-like property, with each domain uniquely binding to separate regions. This feature is likely to endow the ligand with high levels of avidity and selectivity, capable of distinguishing between ion-channel subtypes with precision.

## Defining Screps

**FIGURE 2:**
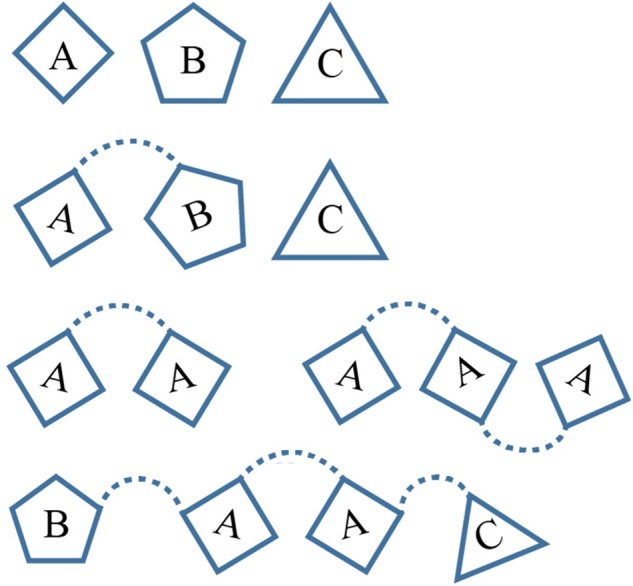
A modular description of multi-domain proteins. The top row demonstrates three separate peptides of different structural domains, while a two-domain and a single domain protein are shown below. The final two rows demonstrate the difference between a “pure domain repeat” and a “combinatorial domain repeat.”

Repetitive protein elements vary greatly in size from individual amino acids and oligo-peptide repeats (Katti et al., 2000), to larger domain repeats, often forming an array of structurally distinguishable complexes (Andrade et al., 2001). It is frequently observed throughout nature that multi-domain proteins may form unique arrangements, changing the gross structural features of the protein, and consequently enabling altered or unique functionality (Apic et al., 2001). Within the protein universe, the descriptive features of each individual protein, as well as multi-domain proteins, can be viewed analogous to linguistics (Gimona, 2006). One could argue the extent to which this metaphor remains applicable, however, its simple use is clearly advantageous when describing modular multi-domain proteins. To elaborate, our understanding behind the meaning of both individual words (functional domains), and their unique combinations (multi-domain proteins), enables the construction of sentences with greater semantic value, i.e., proteins with a higher degree of sophisticated functionality (Scaiewicz and Levitt, 2015). By adopting an analogous approach to the representation of multi-domain protein function, the implementation of a simple and effective method for description may be applied. To illustrate (Figure 2), a series of proteins may be described as [A], [B], [C] reflecting three separate proteins of different domain types, whereas [AB], [C] indicates one multi-domain protein consisting of both domain type [A] and [B] in that order, and one single domain protein [C]. The appearance of such nomenclature will be used throughout this manuscript to outline SCREP domain arrangements, e.g., [AA], [AAA], and [BAAC] are described as a two-domain protein repeat, a three-domain protein repeat, and a four-domain protein with a two-domain repeating core flanked on either side by different domains, respectively.

To accurately describe the unique features of SCREPs, it is necessary to first clarify and establish descriptive terminology. Upon analysis of the domains within SCREP sequences, it becomes clear that there exist two major types. First is the presence of SCREPs that may be classified as having “pure domain repeats,” i.e., [AA], [BBB], [CCCC], etc. Second are SCREPs with “combinatorial domain repeats,” i.e., [BAA], [BBAA], [CCCAB], etc. An additional defining feature of SCREPs is the varying levels of observed “repeat purity,” a term previously defined as the average pairwise sequence identity between the repeating units within each SCREP (Schuler and Bornberg-Bauer, 2016). The approach toward protein classification can stem from both structural and evolutionary perspectives, e.g., the use of automated tools comparing structural geometry such as superimposition of disulfide bridge topology (Mas et al., 1998), defining proteins through their amino acid profiles such as their “disulfide signatures” (Gupta et al., 2004), or simple homology-based annotation transfers that group both the sequence identities and functional similarities between proteins. Here, we employ a combination of cysteine pattern and sequence homology approaches to identify SCREPs, whose multi-domain TR architectures are defined by their cysteine-rich features. This cysteine rich feature further enables a useful approach in which SCREPs may be distinguished from one another. Individual domain types often have shared common features such as typical domain length and overall cysteine content, and this results in a cysteine density which is characteristic of a particular domain type. Therefore, the individual cysteine density calculated for each sequence is also used as a method for clustering SCREPs.

## Identifying Screps

**FIGURE 3:**
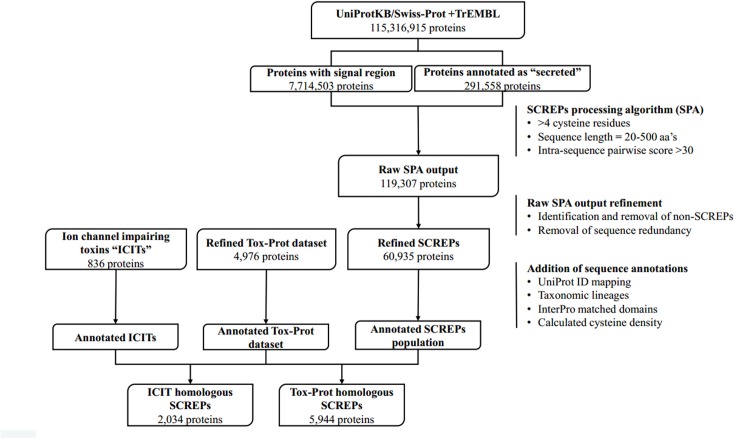
SCREPs data mining pipeline. An overview of the entire SCREPs data mining pipeline, from the generation of SCREP sequences originating from UniProtKB, up to methods of data refinement as well as population and sequence analysis. Beneath each major step is the resulting number of protein sequences with additional details toward the right, outlining important elements within the major steps of the SCREPs processing algorithm (SPA), data refinement, and application of dataset annotations.

The overall data mining process can be broken down into several distinct stages (Figure 3), beginning with the initial extraction of protein sequence data originating from the UniProtKB database; a centralized database of protein sequence information (Apweiler et al., 2004). Data from both Swiss-Prot; the *manually* curated and reviewed sequence space (≈557,00 entries in 2018-04) and TrEMBL; the *automatically* annotated and unreviewed sequence data (≈114 million entries in 2018-04) (Boeckmann et al., 2003) was used. From this, a dataset of proteins likely to be extracellularly secreted is identified and their sequences downloaded, subsequent processing of these sequences using the custom built SCREPs processing algorithm (SPA) generates an initial dataset of SCREP candidates. It is at this point that the raw output of the SPA requires refinement by identifying and removing redundant or non-SCREP sequence data. This process is critical because establishing the best possible starting platform will help prevent analysis errors downstream. Finally, a homology-based search process is implemented; this involves identifying sequence similarities between previously characterized bioactive toxins (with any domain architecture) and the SCREPs dataset. Functional correlations using the resulting list of SCREPs form the basis for a rational approach toward peptide selection for further characterization.

### Identifying Extracellularly Secreted Sequences

In the initial stage of generating the SCREPs dataset it is important to avoid accidental exclusion of any true SCREP sequences. Ideally, the SCREPs identifying algorithm would be applied directly to the total UniProtKB database (≈114 million sequences as of May 2018), however, processing a dataset of this size is a computationally very intensive. Therefore, reducing the dataset size is necessary for making this task more feasible. To capture all potential SCREPs, appropriate filters to extract secreted proteins must be applied, the first of which is to exclude intra-cellular proteins. However, identifying proteins that are extracellularly secreted encounters many inherent difficulties. As previously mentioned, secreted proteins contain a signal sequence region, and the primary tool used to identify the presence of this region is SignalP (Bendtsen et al., 2004). Despite SignalP 4.0’s capabilities of distinguishing between signal peptides and transmembrane regions (Petersen et al., 2011), it is still unable to differentiate between regions associated with intra- and extracellular translocation. The current difficulties and limitations of identifying secreted proteins is an important point of consideration, particularly when interpreting downstream results, i.e., the potential presence of non-extracellularly secreted proteins within the dataset – i.e., false positives.

Conversely, there is a need to consider sources of false negatives in the initial dataset. UniProt facilitates the search of sequences that have an identified signal region, which currently contains ≈8 million sequences. However, applying SPA to only sequences containing this signal region is not sufficient in capturing all SCREPs. This is due to the occurrence of uploaded protein sequences derived from native material wherein the signal region has undergone proteolytic cleavage prior to sequencing. Often in this situation, the uploaded sequences have been manually reviewed and annotated, allowing for a simple search of proteins with a “secreted” subcellular annotation, which currently results in ≈300,000 sequences. At the time of applying this data mining process, the signalP dataset contained 7.7 million sequences, and the total “secreted” dataset held 291,558 sequences. Combined, the starting platform in which SPA was applied contained just over 8 million protein sequences.

### SCREPs Processing Algorithm (SPA)

The existing population of TR, multi-domain protein architectures is difficult to define, especially proteins with highly diverged sequences between domain repeats. Identifying the presence of TR proteins has proven to be a difficult task that is not easily accomplished; several different approaches have been presented with inherent strengths and weaknesses. Past attempts at estimating the existing population of TR proteins within the Swiss-Prot database identified ≈13–20% occurrence (Marcotte et al., 1999; Pellegrini et al., 2012). Many bioinformatics tools and resources have previously been developed to address the difficult task of analyzing TR proteins, and RepeatsDB is one such database that provides structural annotation and classification (Di Domenico et al., 2014). The primary tool later developed to assist with the annotation of RepeatsDB, ReUPred, was designed to receive a target protein structure as input and output predicted repeat unit positions, enabling the application of repeat classification schemes (Hirsh et al., 2016). However, this approach is only valuable if such structural information is available. Due to the enormous disproportion between available structural information and sequence data, the application of sequence-based identification methods is of greater value with regards to bio-discovery efforts then the characterization and classification of previously studied structures. When attempting to identify the presence of TR units within the TrEMBL sequence space, the lack of knowledge surrounding these entries, particularly the absence of structural data, forces the application of methods based exclusively on primary sequence information.

Presented here is the SCREPs processing algorithm (SPA); a custom-built in-house tool developed specifically to address the unique primary sequence features found within SCREPs. Prior to the application of the SPA tool, identification and removal of any signal sequence region that may exist within the protein is essential. Naturally, the overall aim of identifying repeating units applies only to the mature protein sequence. The first stage of the SPA initially processes each sequence to identify suitable SCREP candidates based upon simple sequence features. This involves restricting the input sequence size to between 20 and 500 aa’s in length; the minimum is set to avoid possible false identification of small non-domain repetitive elements, and the upper limit is established to avoid much larger proteins, e.g., transmembrane receptors. Additionally, to identify sequences that would contain at least two independently folded DRDs requires a minimum of 4 cysteine residues, i.e., the formation of at least one disulfide-bond per domain.

Following these filtering steps, each individual sequence is divided into a series of amino acid segments that may vary in length. A number (*n*) of amino acids is counted from the N-terminus and used to divide the protein sequence into two segments. These two segments are used for pairwise sequence alignment by *blastp* (Madden et al., 1996). The division site is then increment by another *n* amino acids to generate two new segments to be compared, the procedure is repeated until the protein sequence has been exhausted. Optimization of the *n*-variable was based on initial testing, offsetting performance and accuracy, resulting in a suitable compromise of 10 aa’s in length. The positive identification of a significant alignment between any of the pairs of segments indicates the potential existence of a tandem repeat architecture. The standard substitution matrix BLOSUM62 (Henikoff and Henikoff, 1992) is used to calculate intra sequence alignment scores, and a threshold value determines whether or not an identified alignment is classified as a strong or weak “hit”; if the alignment score >30 the self-aligned sequence is considered a strong hit, while anything <30 is considered weak. The main focus of this work is on the refinement and analysis of the strong hits only. Overall, the application of the SPA on all 8 million mature protein sequences containing both a signal sequence region and annotated as “secreted” resulted in a preliminary SCREPs dataset of 119,307 sequences (1.5%).

### Refinement of Raw SPA Output

**FIGURE 4:**
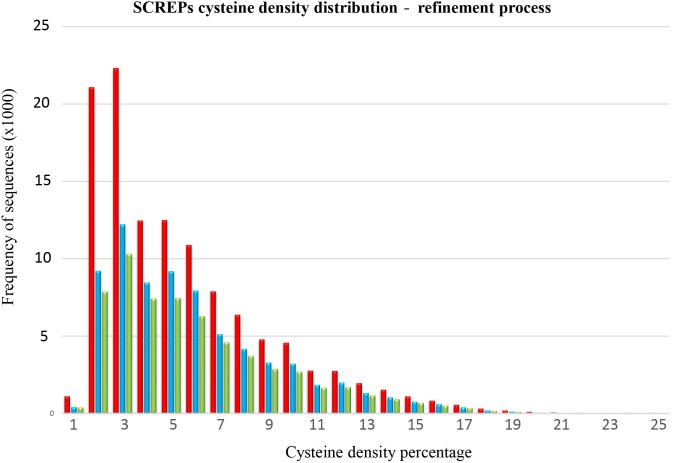
SCREPs cysteine density distribution. An overlay of the cysteine density distribution of the three intermediate SCREP datasets demonstrating the refinement process, one; the raw output of the SCREPs processing algorithm “SPA” (red), two; after the removal of identified non-SCREP domains (blue), and three; the final non-redundant refined SCREPs dataset processed using cd-hit (green).

Certain considerations must be included due to the current sequence processing limitations used to identify extracellularly secreted proteins. This will inevitably result in the contamination of the raw SPA sequence output data with non-secreted cysteine rich proteins. At this preliminary stage, a strict systematic approach toward isolating non-SCREP sequences has yet to be developed, resulting in a process that requires a degree of manual curation. Fortunately, the defining disulfide-rich nature of SCREPs provides a common reference point in which to build an efficient clustering method for DRD containing proteins. To achieve this, we clustered sequences based on their overall cysteine density (relative to the entire SCREP sequence). This approach revealed the existence of a bimodal distribution of cysteine density. Sequences with 2–3% cysteines contribute to over one third of the resulting SPA output (Figure 4). The domain analysis performed on this significant 2–3% cysteine density range led to a deeper investigation to determine the nature of these sequences. This revealed a high frequency of cytochrome C domains (>15,000 sequences), as well as several thousand thioredoxin-type folds, demonstrating the presence of non-SCREP proteins within the raw output. The role of two cysteine residues in cytochrome C that are involved in the interaction of various iron containing heme groups is well known (Meunier et al., 2004), and the abundance of multi-heme interacting cytochrome proteins (Breuer et al., 2015) directly explains why they were identified by the SPA. Furthermore, two key cysteine residues within thioredoxin are essential for the activity of a disulfide/dithiol site, essential in facilitating reduction of other proteins (Arner and Holmgren, 2000). These two protein domains provide excellent examples where their cysteine residues are not primarily involved in formation of disulfide bonds which direct and stabilize the protein structure. Therefore, these proteins lack a multi-domain architecture of independently folded DRDs, the distinguishing structural feature of SCREPs. Removing sequences where a few catalytic or metal coordinate cystines are incorrectly flagged as potential disulfide bonds led to the removal of a significant amount of data; 47,170 non-SCREP sequences were removed, reducing the raw SPA output by 39.5% and leaving a refined dataset of 72,137 SCREP candidates.

An additional step within the SCREPs refinement process is the identification and removal of sequence redundancy. To determine what level of redundancy may exist between sequences within the entire SCREPs population (119,307 sequences), we used cd-hit (Li et al., 2001) to cluster sequences with >99% identity. This approach identified 98,628 unique clusters, and indicates that the SPA raw output contains ≈17.3% (20,679 sequences) of redundant data. Similarly, cd-hit was also applied with the same threshold to the refined dataset (72,139 sequences), resulting in 60,935 unique clusters and reducing the refined SCREPs dataset by ≈15.5%. The reduction of redundant sequences within the refined SCREPs dataset appears proportional to each cysteine density cluster, suggesting no particular bias with a general sequence redundancy across the population. It is worth noting that the application of a 99% threshold will naturally remove all sequence duplicates, as well as many highly similar sequences. However, its accuracy may decline with smaller sequences where even one amino acid substitution may lead to a shared sequence identity <99%. Similarly, the lowering of this threshold may result in the accidental removal of large sequences that may in fact have a greater difference in residues, deeming the cluster not redundant and in fact valid. The above procedure was largely achieved by manual inspection and an improved systematic approach toward identification and removal of non-SCREP sequences will greatly improve the throughput of the data mining process and is the subject of further research.

## The Screp Universe

**FIGURE 5:**
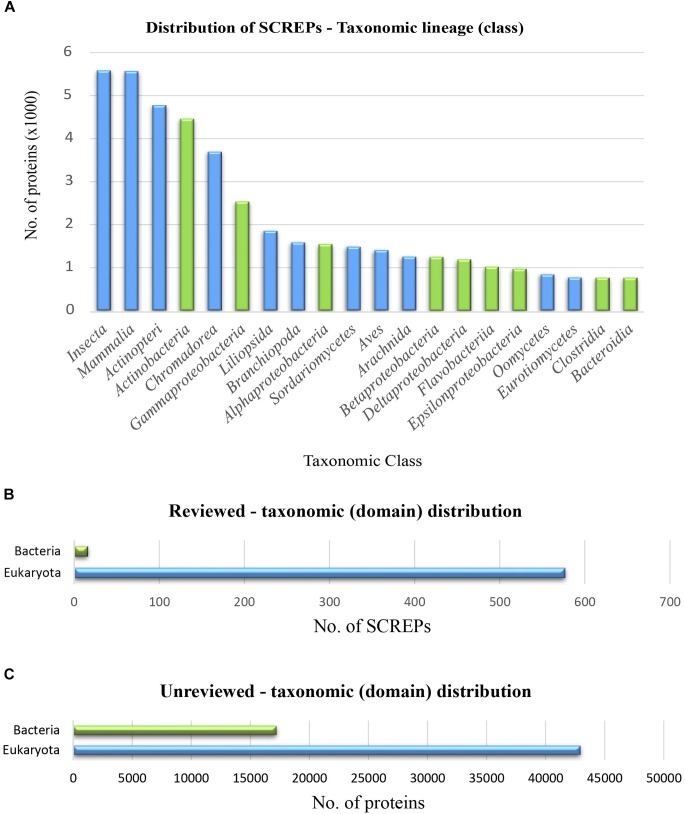
Taxonomic distribution of SCREPs. **(A)** Distribution of the 20 highest occurring taxonomic classes of SCREPs, blue indicating eukaryotic members and green indicating bacterial. **(B)** The total frequency of reviewed SCREPs within their respective taxonomic domains. **(C)** The total frequency of unreviewed SCREPs within their respective taxonomic domains.

Proteins within the refined SCREPs dataset, represent a great level of diversity with 166 unique taxonomic classes. A distribution of these major classes demonstrates the possible central role this novel protein architecture may play, bridging distantly related organisms between two distinct domains of life. A significant portion of SCREPs are clearly found within both bacteria (green) and eukaryotic organisms (blue) (Figure 5A). The current study of proteins within eukaryotic organisms clearly dominates the 1% of reviewed SCREPs from the Swiss-Prot database (Figure 5B). The remaining 99% of unreviewed sequences seen within (Figure 5C), likely demonstrates a more accurate reflection of the reality in which their existing proportions may sit. This dramatic disproportion is likely a result of past difficulties in obtaining sufficient protein material for detailed studies.

Different functional relationships are likely to exist between SCREPs within particular taxonomic groups, leading to clusters of specific biological roles throughout. So far, we have already observed such roles as protease inhibition and ion-channel modulation from venomous and parasitic species. However, there still appears to be a large amount of SCREPs observed within non-venomous and parasitic classes also, these are particularly apparent within the vertebrate classes Mammalia, Actinopterygii, and Aves (Figure 5A). Further characterization of this refined dataset is necessary in order to begin delineating the domain architectures that are characteristic of such classes. This would result in a more detailed picture of these proteins, improving the basis for functional hypotheses. For the purpose of this work, however, our focus will remain primarily fixed upon the analysis of SCREPs within venomous/parasitic organisms, identifying regions of the SCREPs sequence space that may provide guidance toward identifying SCREPs with ion-channel activity.

### The SCREP Structural Landscape

Given the apparent taxonomic diversity among SCREPs, we further investigated whether this level of diversity extends to the structural landscape as well. Due to the nature of this dataset, there is little to no structural information available for these proteins, therefore, the InterPro resource was used (Apweiler et al., 2000). The identification of domains from unknown protein sequences can be searched for using InterProScan, a tool that matches all possible sequence regions to previously defined domain profiles that exist within the InterPro consortium (Quevillon et al., 2005).

**FIGURE 6:**
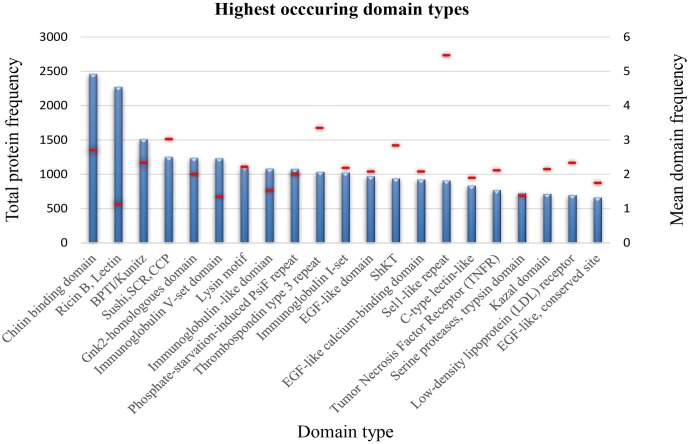
SCREP structural diversity. Distribution of the 20 highest occurring domain types identified by InterPro within the refined SCREPs population, showing frequency of proteins within an individual domain type (blue), and the mean domain frequency (red line overlay).

Using InterProScan, 98.4% of the sequences within the refined SCREPs dataset were identified as having matching domain sequence features to at least one of the databases within the InterPro consortium. The resulting output of InterProScan after processing thousands of multi-domain protein sequences generates an immense amount of data to interpret and a manual approach toward such analysis is very limited. Using a custom, in-house developed tool to summarize domain distributions showed that SCREPs are by no means limited to the already identified Kazal and ICK domains. Surprisingly, these folds were not among the top 20 most abundant SCREP folds, which comprise a large diversity of structural domains (Figure 6). Plotting the number and mean number of domains per protein also revealed a significant presence of complex multi-domain protein sequences within the SCREP dataset. Domain types with a mean value approaching one, likely indicate the presence of non-repeating elements found within “combinatorial domain repeats,” for example [BAA], with B reflecting domain types such as Ricin B, lectin (Figure 6). There are several examples of significantly high levels of domain type frequency, with quite noticeable proteins including; chitin binding, bovine pancreatic trypsin inhibitor (BPTI)/Kunitz, Sushi, thrombospondin type 3 repeat, and the Sel1-like repeat domains. Interestingly, for the chitin binding domain and Sel1-like repeat domains, the extent of such high levels of unit repetition reaches up to 8 and 12 repeats, respectively. From this preliminary analysis, it is quite clear that the SCREPs universe indeed contains a large amount of both taxonomic and structural diversity. Future efforts toward improved characterization of this dataset will likely reveal valuable insights, through a detailed correlation between taxonomy and the associated domain architectures, allowing us to develop a better picture of the SCREPs universe.

## Identifying Putative Bioactive Screps

With an understanding of both the size and diversity of the SCREPs dataset, the question emerges of how this potentially useful resource may be fully utilized to identify functionally active peptides. To identify SCREPs that are likely to demonstrate ion-channel activity, it is necessary to narrow our focus toward smaller subpopulations of the database. To achieve this, we employed additional, widely available bioinformatics tools and resources to refine our database. We first used a dataset of toxins and proteins derived from venomous organisms (Tox-Prot) (Jungo and Bairoch, 2005). The continued efforts made in annotating this dataset (Jungo et al., 2012), provide a well-established platform which represents an excellent reference of toxin structure and function.

A more sophisticated approach of interpretation is essential when attempting to accurately correlate any structure-activity-relationship, in particular, a cause for caution is necessary in avoiding inaccurate inferences. The Tox-Prot dataset downloaded for use in this comparative analysis contained a total of 6,722 protein sequences (February 2018), but consisted of approximately of this dataset contains sequence fragments that were excluded. Further refinement of this dataset by cd-hit to remove any redundant sequences reduced the original Tox-Prot dataset by ≈26%, resulting in a non-redundant and fragment-free dataset containing 4,976 venom protein and toxin sequences.

The first and probably most valuable relationship to observe is the presence of any significant pairwise alignments identified using BLAST. Local alignment of the mature sequences from both SCREPs and Tox-Prot, was performed using the standard blastp program with an *e*-value cut off of 10^-6^; a value set in order to identify alignments of moderate to high similarity. The resulting dataset of Tox-Prot SCREPs (TP-SCREPs) contained a considerable 9,925 alignments, matching 494 Tox-Prot sequences with 5,944 SCREPs. The BLAST results identified 25 SCREP sequences that were also present within the refined Tox-Prot dataset. Confirming the functionality of our approach, these include the previously mentioned SCREPs such as DkTx, CpTx-1, as well as Hi1a and its 3 additional variants; Hi1b-d. Within the remaining 25 SCREP sequences shared between datasets, the identification of a few putative SCREPs is observed, these include two spider derived peptides; Pn1a from *Phoneutria nigriventer* with an apparent double thyroglobulin type domain, U19-barytoxin-Tl1a, a double Kunitz type peptide isolated from *Trittame loki*, as well as two double WAP type snake derived peptides; Waprin-Phi1 and Waprin-Enh1, isolated from *Philodryas olfersii* and *Pseudoferania polylepis*, respectively.

**FIGURE 7:**
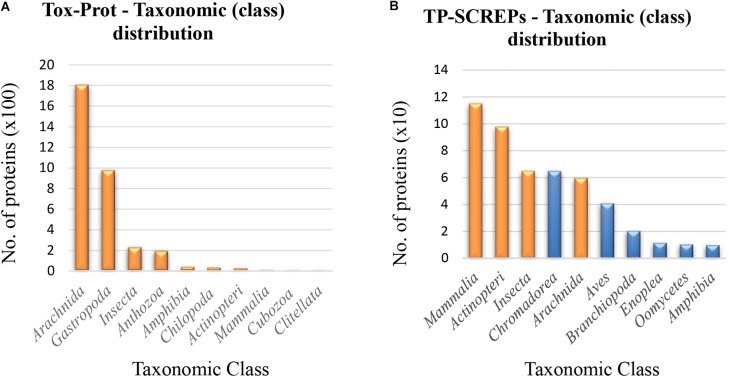
Taxonomic distribution of Tox-Prot and TP-SCREPs. **(A)** The taxonomic distribution of classes within the refined Tox-Prot dataset. **(B)** The taxonomic distribution of SCREPS that display homology with the refined Tox-Prot dataset “TP-SCREPs,” orange indicating the overlap of taxonomic class with Tox-Prot.

The taxonomic distribution observed within the Tox-Prot dataset (Figure 7A) contains a low level of diversity, with the majority of proteins originating from the *Arachnida* and *Gastropoda* classes. Surprisingly, the distribution of TP-SCREPs does not reflect the same bias toward these taxonomic groups (Figure 7B), with a significant number of proteins identified from both overlapping classes (orange in Figure 7B), as well as separate classes (blue in Figure 7B). The more uniform taxonomic distribution seen in TP-SCREPs is likely a result of the data driven approach toward identification, including many uncharacterized proteins derived from transcriptomic data rather than just the obtainable venom extract. The TP-SCREPs that display a shorter evolutionary divergence with the correlating Tox-Prot organisms may have an increased probability of sharing functionality, and selection of SCREPs between overlapping taxonomic regions is likely to improve the accuracy of activity predictions. Further analysis of these sequences is likely to yield the identification of bioactive SCREPs with a higher level of confidence.

## Identifying Putative Ion-Channel Impairing Screps

With the likely occurrence of many additional examples of bioactive SCREPs, we next examined the SCREPs database for peptides with putative ion-channel activity. The Tox-Prot dataset contains a significant number of useful protein sequences with experimentally supported functional annotations. The Tox-Prot dataset was, therefore, used as the basis for generating the dataset of ion-channel impairing toxins (ICITs). The ICIT dataset was generated from the Swiss-Prot database by keyword searching for the terms “toxin” or “venom” within all reviewed proteins of the Metazoan kingdom. Finally, a search to identify which of these sequences are associated with “ion channel impairing” functionality supported with experimental evidence, produced the final ICITs dataset containing 890 unique sequences. This dataset can be further divided based upon their associated ion-channel interactions, allowing for the possibility of guiding the selection of ion-channel specific homologous SCREP sequences. Like the refinement of all previous datasets, cd-hit was applied which generated a dataset of 836 non-redundant ICITs.

To generate local alignments, blastp was used with an *e*-value cut off at 10^-6^, and this resulted in a total of 3,391 significant local alignments (>35 bit score), with 2,034 SCREPs (ICIT-SCREPs) from 50 unique ICITs. Similar to the BLAST analysis between the Tox-Prot data, a few overlapping sequences were identified which revealed the presence of three SCREPs already within the ICIT dataset, including DkTx, Hi1a, and a precursor protein from the anemone *Urticina grebelnyi* (Osmakov et al., 2013). While the presence of DkTx and Hi1a again served to validate our approach, the addition of this third sequence represents a new challenge for the interpretation of SCREP data. This identified SCREP comprises 4 domains, however, each domain is flanked by a propeptide region resulting in three unique but structurally similar individual toxins, only one of which has been identified to interact with the ion channel ASIC3 (π-actitoxin-Ugr1a). This third protein represents just one of a likely group of possible multi-domain sequences in which post translational modification proteolytically separates the domains, preventing any sort of SCREP like structural formation (i.e., those leading to a potential bivalent mode-of-action). It is likely that many protein sequences within this dataset may have evolved to be post translationally cleaved, resulting in multiple separate functional units. However, this diverges from the intended goal of studying multivalent interactions that drive potentially unique pharmacology. Therefore, it is only appropriate to attempt to identify, and remove any protein sequences that may have an inherent cleavage site between domains. This causes additional considerations, requiring both careful interpretation and further bioinformatics optimization to accurately identify and distinguish between such sequences.

### Diversity of ICIT-SCREPs

**FIGURE 8:**
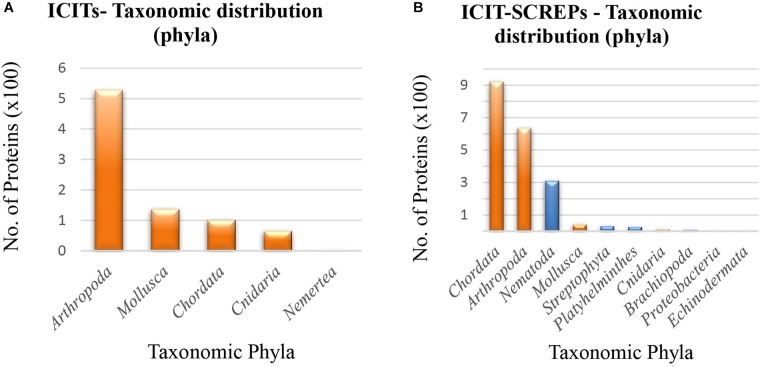
Taxonomic distribution of ICITs and ICIT-SCREPs. **(A)** The taxonomic distribution of phyla within the refined ICIT dataset. **(B)** The taxonomic distribution of SCREPS that display homology with the refined ICITs “ICIT-SCREPs,” orange indicating the overlap of taxonomic phyla with the ICITs.

Similar to the taxonomic diversity observed within the Tox-Prot dataset, the ICIT proteins also display a narrow distribution containing only five different phyla (Figure 8A). Dominating these representatives is the arthropods, distantly followed by Mollusca, Chordata, and the Cnidaria. In contrast, the ICIT-SCREPs data encompasses a greater taxonomic diversity, with 20 distinct phyla observed. The major contributing phyla within the ICIT-SCREPs dataset are; Chordates (45.5%), arthropods (31.4%), and Nematodes (15.4%). While the abundance of the arthropod-derived ICITs clearly influences its reappearance within the ICIT-SCREPs data (Figure 8B, orange), we also observed a significant frequency of pairwise alignments identified from the more distantly related Chordata, with classes such as Mammalia, Actinopterygii and Aves. To address the curious presence of these proteins, further investigation into the shared sequence features and alignments is required. However, in search for related functionality, the analysis of SCREPs from overlapping classes, as well as more closely related ones such as Chromadorea, Branchiopoda, and Enoplea will likely yield better results (in terms of presence of ion-channel impairing bioactivity). Regarding domain type distribution, the BPTI/Kunitz type fold dominated the ICIT-SCREPs, while the occurrence of other folds frequently observed within the ICITs such as ICK domains was much less apparent. This is possibly a reflection on the level of sequence conservation observed between Kunitz type domains, enabling identification using simple BLAST methods. By comparison, the low level of ICK type folds found within the entire SCREPs dataset indicates that the Kunitz domain is quite abundant throughout.

### Analysis of ICIT Homologs

Described here, are details relating to 50 ICITs out of 836 that display significant levels of sequence similarity with SCREPs. Additional parameters that were used to reduce dataset size include removal of poorly annotated ICITs, and selection of ICITs that display multiple SCREP matches. The small size of this dataset provides an excellent opportunity to manually review these toxins, outlining their taxonomic and structural features, as well as their pharmacology, focusing on their ion-channel interactions. Although there may be many cases whereby the homologous SCREPs (ICIT-SCREPs) do not share functionality, the functional analysis of these 50 ICITs may still provide valuable insight toward the SCREPs that do. Naturally, further experimental investigation is required to conclusively determine which ICIT-SCREPs out of the 2,034 homologous sequences may share ion-channel activity. However, simple bioinformatics analysis is the first step toward identifying possible ion-channel modulating SCREPs, reducing the number of peptides required for any functional screening endeavors.

In contrast to the heavily disproportionate taxonomic distribution of the entire ICIT dataset, the overall distribution of these 50 homologous ICITs begin to take a more uniform shape, reflecting a similar distribution to that observed within SCREPs. The phyla observed within these 50 ICITs include an even split of originating organisms from both arthropods and chordates, collectively making up 77.5% of the dataset, with the remaining ICITs derived from the Cnidaria (18.4%), and Mollusca (4.1%). As previously mentioned, the predominant domain type within the ICIT-SCREPs dataset is the BPTI/Kunitz domain, and unsurprisingly this domain constitutes the majority (64%) of the homologous ICITs. A bias toward this domain type may possibly be due to either the method used in this approach, or simply just a direct reflection of the abundance of Kunitz domains within the SCREPs data. Several other shared domain types exist which include scorpion long chain toxins (cystine-stabilized a/β fold: CSaβ), cysteine-rich venom proteins (CRVP), PLA_2_, as well as a few representatives from knottin-type scaffolds. These peptides display varying degrees of observed functionality toward their ion-channel targets in terms of potency, selectivity, and levels of supporting experimental evidence. In terms of their ion-channel targets, most of these toxins (51%) inhibit voltage gated potassium channels (*K*_V_), an even amount (10.2%) target both voltage gated calcium and sodium channels (Ca_V_, Na_V_), similarly, an equal amount (6.1%) of toxins target both ryanodine receptors (RYR) and transient receptor cation channels of the vanilloid group (TRPV), and finally (8.2%) target acid sensing ion channels (ASICs). It is possible that the common occurrence of homologous ICITs found targeting the homotetrameric *K*_V_ channels may indicate a potential bias toward SCREP interactions due to their multivalent ligand behavior. The nature of SCREPs with internally homologous domains may perhaps favor binding with molecules that display structural symmetry, enabling multivalent interactions to occur across identical subunits such as that observed within potassium channels (Doyle et al., 1998).

### ICIT and SCREP Sequence Alignments

**FIGURE 9:**
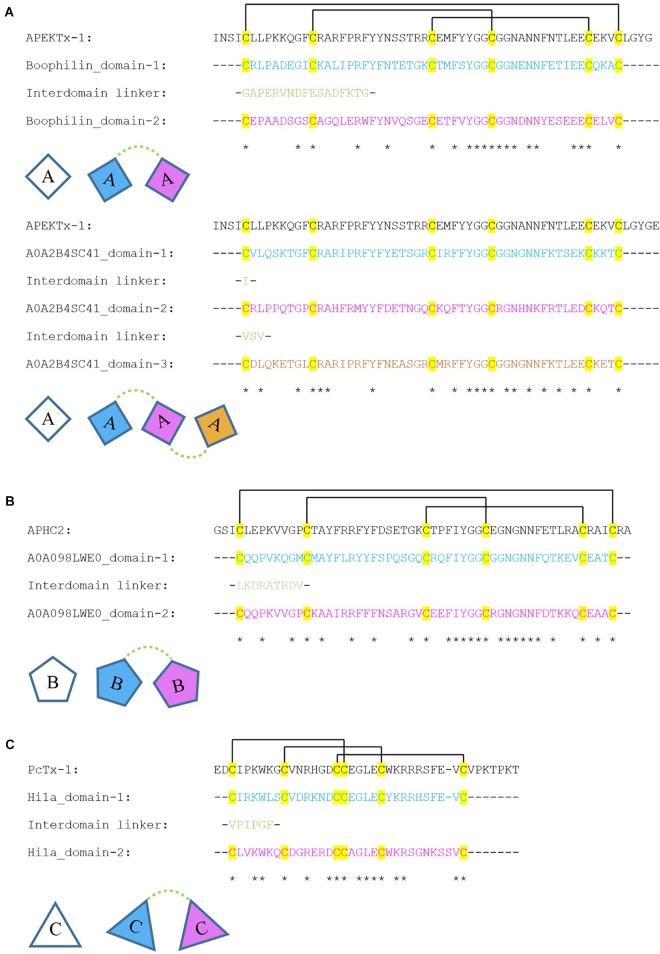
Alignment of single domain toxins with multi-domain SCREPs. These series of alignments are between three single domain ion channel impairing toxins; **(A–C)** with the domains of four SCREPs. The disulfide connectivities are indicated by lines between cysteine residues. The top alignment is between APEKTx1 **(A)**, Boophilin [AA], and the novel three-domain SCREP sequence; A0A2B4SC41 [AAA]. The middle alignment is between APHC2 **(B)** and the two domain SCREP sequence; A0A098LWE0 [BB]. The bottom alignment is between PcTx-1 **(C)** and the double ICK SCREP Hi1a [CC]. All uncharacterized proteins have been labeled with their associated UniProt identifiers.

To more closely examine putative ion-channel modulating SCREPs, we selected high-similarity hits to three ICITs with high levels of experimental annotation that represented two different fold types and three different ion-channel targets (Table 1). These high quality ICITs were used to probe the sequence similarities within SCREP domains by first manually reviewing the homologous ICIT-SCREPs, taking into consideration multiple features such as primary sequence alignment scores, proximity of taxonomic relationships, as well as overall SCREP domain architecture; preferencing SCREPs with “pure domain repeats”. Presented here are details of four sequence alignments between these three ICITs (Figure 9), with half the alignments occurring in previously studied SCREPs with known functionality. The fact that this process has resulted in the identification of known bioactive SCREPs, partially validates our approach. Complementary to these alignments, we also provide two additional examples of completely novel SCREPs with putative ion-channel activity. Since these are completely uncharacterized peptides, they have been labeled with their respective UniProt identifiers.

**Table 1:** Selection of relevant ion channel impairing toxins (ICITs).

ICIT (UniProt ID)	Length	Domain/fold type	Associated ion channel	Organism	Number of SCREP alignments (Unique SCREPs)
APEKTx1 (P86862)	65	BPTI/Kunitz	rKv1.1/KCNA1	*Anthopleura elegantissima*	234 (117)
APHC2 (C0HJF4)	56	BPTI/Kunitz	TRPV1	*Heteractis crispa*	61 (34)
PcTx1 (P60514)	40	Knottin, omega toxin-like	ASIC1a	*Psalmopoeus cambridgei*	8 (4)

*This list contains 3 ICITs out of the 50 peptides identified as sharing significant sequence identity With SCREPs. Supplemented With each sequence Are relevant details outlining peptide length, the recognized domain/fold type, their interacting ion channels (supported With experimental evidence), the organism of origin, and finally, the total number of alignments identified in the original BLAST search. These were chosen based on the level of annotations available which are supported with a high degree of experimental evidence*.

The first alignment (Figure 9A) is between the ICIT APEKTx1, a 65 residue BPTI/Kunitz type peptide with bifunctional serine protease activity and selective K_V_1.1 inhibition [IC_50_ 0.9 ± 0.1 nM; (Peigneur et al., 2011)] isolated from the sea anemone *Anthopleura elegantissima*. The SCREP with the highest scoring pairwise alignment with APEKTx1 that originates from a venomous or parasitic organism, is the previously studied Boophilin-H2 peptide, a multifunctional two-domain BPTI/Kunitz SCREP (Macedo-Ribeiro et al., 2008). Further investigation of Boophilin-H2 may reveal a bi-functional role similar to APEKTx1, possibly acting as both a serine protease and ion channel inhibitor. The second alignment in (Figure 9A) is between APEKTx1 and a three-domain SCREP (A0A2B4SC41) originating from the scleractinian *Stylophora pistillata*, which is also the only similar cnidarian SCREP. All three domains contain zero gap openings and a sequence identity of between 43 and 62% with APEKTx1, suggesting the possibility of shared functionality. The linker regions between each domain are very short, containing a single isoleucine residue between domains 1–2, and a tripeptide linker; valine-serine-valine, between domains 2–3. Assuming no proteolytic separation of domains, this may represent a very interesting three-domain SCREP with a potential trivalent ion channel interaction.

Similar to APEKTx1, APHC2 is another sea anemone-derived Kunitz peptide isolated from *Heteractis crispa* with dual functionality as both a serine protease inhibitor and ion-channel modulator that reversibly inhibits mammalian TRPV1 channels (Andreev et al., 2012). The highest scoring SCREP alignment with APHC2 belongs to the second domain of an uncharacterized, 119 residue, putative toxin with two Kunitz domains isolated from the venomous sea snail *Turridrupa cerithina* (A0A098LWE0) (Figure 9B). Additionally, this peptide displays 56% sequence identity with a novel 113 residue peptide from *Gemmula speciosa* (A0A098LW49). These two unstudied SCREP sequences represent exciting and new possible examples of bivalent ion-channel ligands.

The third ICIT alignment investigated was using PcTx-1 (Figure 9C). Isolated from the venom of tarantula *Psalmopoeus cambridgei* (Escoubas et al., 2000), this is an extensively studied peptide that forms an ICK type structure (Escoubas et al., 2003), and functionally inhibits ASIC1a and potentiates ASIC1b (Chen et al., 2005, 2006). The four SCREP alignments that share a significant level of sequence identity with PcTx-1 are the double-ICK peptide Hi1a and its three variants, Hi1b–d. Both domains of Hi1a demonstrates a high level of similarity with PcTx1, with domain 1 displaying 71.4% sequence identity (Figure 9). As previously discussed, Hi1a is a noteworthy ion-channel modulating SCREP that has been shown to interact with ASIC1a (Chassagnon et al., 2017), and the conserved residues between these two toxins therefore clearly contain critical regions required for ASIC1a interactions. The identification of Hi1a, a prominent SCREP that demonstrates a unique bivalent interaction with ion channel ASIC1a, further promotes this approach as a valid method toward the identification of additional SCREP ligands.

The unique TR multidomain structure of SCREPs is likely to be responsible for their observed multivalent interactions. The investigation of SCREP ligands with multiple binding sites on symmetrical targets may lead to new insights on the role of bivalency in ion-channel pharmacology. Previous observations from DkTx and Hi1a, suggest that these features may enhance the selectivity of these ligands, capable of differentiating between ion-channel subtypes. Additionally, the combined interactions between multiple binding sites may result in increased levels of ligand avidity, with desirable pharmacological consequences. The formation of a tripartite complex between DkTx, TRPV1, as well as phospholipid interactions, have proven useful in the structural elucidation of this ligand receptor complex (Gao et al., 2016). Additionally, the observation of both DkTx and Hi1a, suggests that these multidomain ligands appear to retain unique functionality even when separated into their individual units. Therefore, the analysis of just one two-domain SCREP, can potentially provide 3 unique interactions in which to investigate; that of the first and second domain, as well as the full-length peptide.

## Screps, Future Perspectives

The data driven methods discussed above, provide a taxonomically unbiased approach toward the investigation of SCREPs. The practical application of our data mining and analysis pipeline, illustrates an excellent opportunity in which to diverge from repeating past methods used in bio-discovery efforts (Mobli et al., 2017). By deviating from this frequently used historical path, a potential increase in the novelty of newly found ligands such as SCREPs may begin to emerge, leading to the observation of highly unique ion-channel pharmacologies such as the bivalent modulatory effects of DkTx and Hi1a (Bohlen et al., 2010; Chassagnon et al., 2017). Hopefully this overview will provide sufficient details outlining both the features of this new source of biomolecules as well as a practical data mining approach necessary for further investigation. Interestingly, recent similar workflows aimed at the investigation of DRPs provide a very applicable demonstration, describing the implementation of large scale production and screening techniques of cysteine dense ion channel ligands (Correnti et al., 2018). Furthermore, recent advances in both recombinant DRP production as well as techniques in obtaining correct disulfide bond connectivities (Berkmen, 2012; Klint et al., 2013) provide more affordable methods that result in improved experimental accessibility toward the investigation of SCREPs.

## Conclusion

The investigation of ion-channel activity depends greatly upon the use of functionally active molecular tools, the interaction of which provides valuable insights into these incredibly complex macromolecules. Animal toxins provide an excellent source of useful ion-channel ligands, and the discovery of novel bioactive ligands is a key component in the study of ion-channel pharmacology, necessary for the therapeutic development of new molecules aimed at treating an array of problematic channelopathies. SCREPs provide an exciting new source of structurally unique proteins with a high level of both taxonomic and structural diversity. Periodic sampling of the SCREP sequence space indicates an increase of approximately 21% of the total dataset size within just 12 months, suggesting this to be an expanding resource.

Presented here, is a detailed analysis of SCREP sequences identified when using the data mining pipeline which we have developed. An effort toward providing a comprehensive description of the SCREPs data mining pipeline will hopefully supply the reader with the necessary details to further explore this novel resource. Despite several bioinformatic challenges that exist, we are still able to confidently identify many putative SCREP sequences that have great potential in displaying unique structure and pharmacology. The analysis of venom derived SCREPs provides an excellent approach in the identification of potentially bioactive peptides, likely to contribute to our understanding of the structural biology, function, and possibly even diversity of ion channels.

## Author Contributions

MiM generated the data presented and performed the data analyses. MeM and MiM designed and developed the bioinformatic tools used with input from EU. EU and MeM conceived of and directed the research with input from MiM. MiM wrote the manuscript with input from all authors.

## Conflict of Interest Statement

The authors declare that the research was conducted in the absence of any commercial or financial relationships that could be construed as a potential conflict of interest. The reviewer IV and handling Editor declared their shared affiliation at the time of review.
